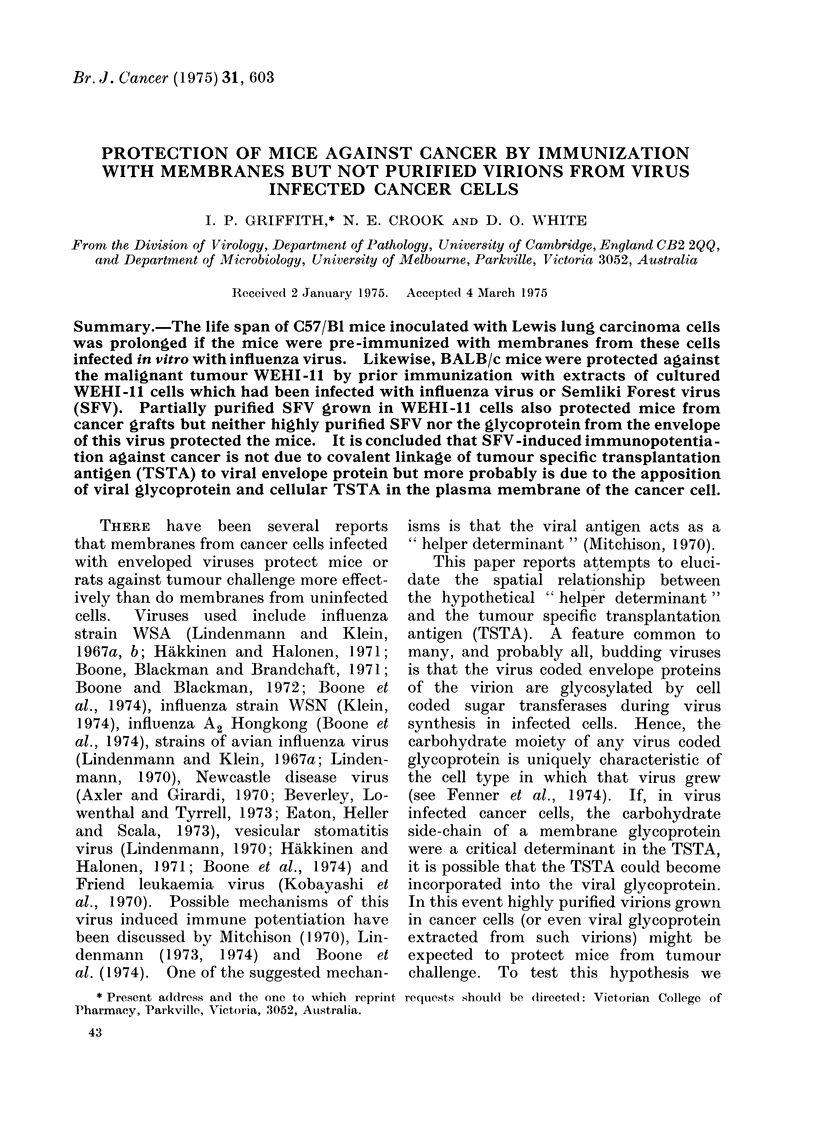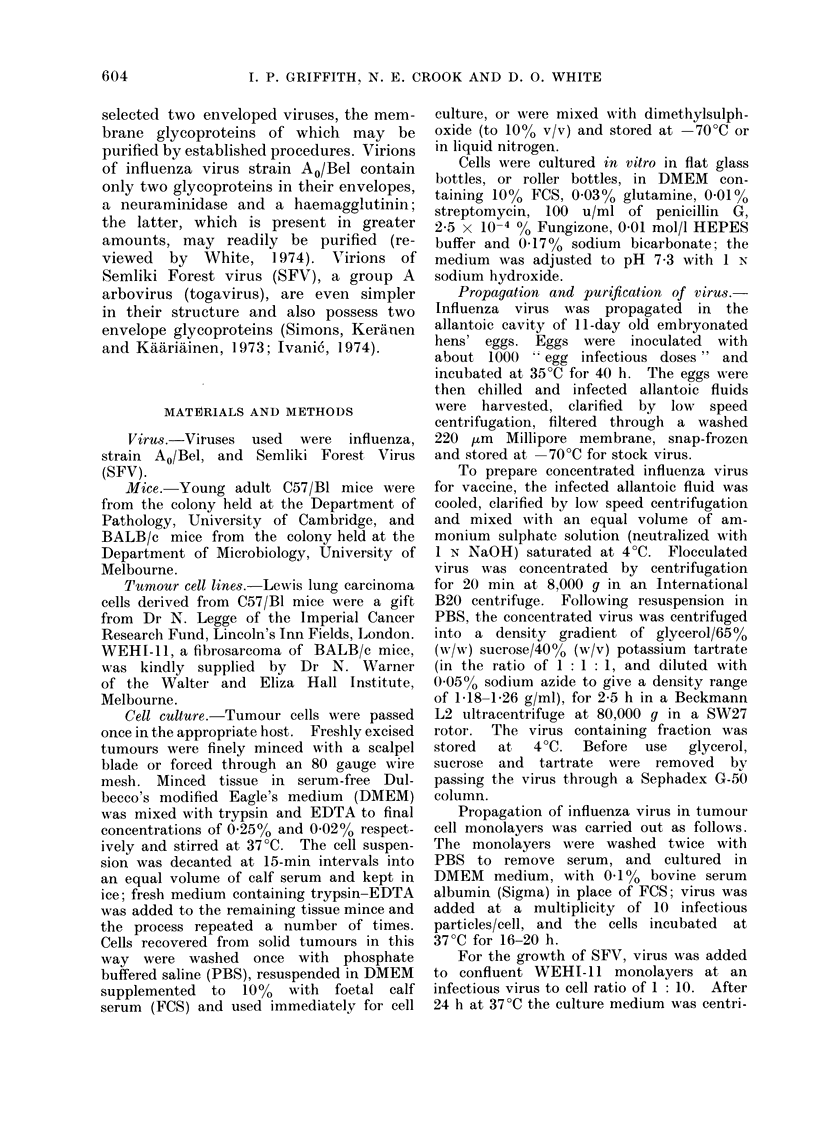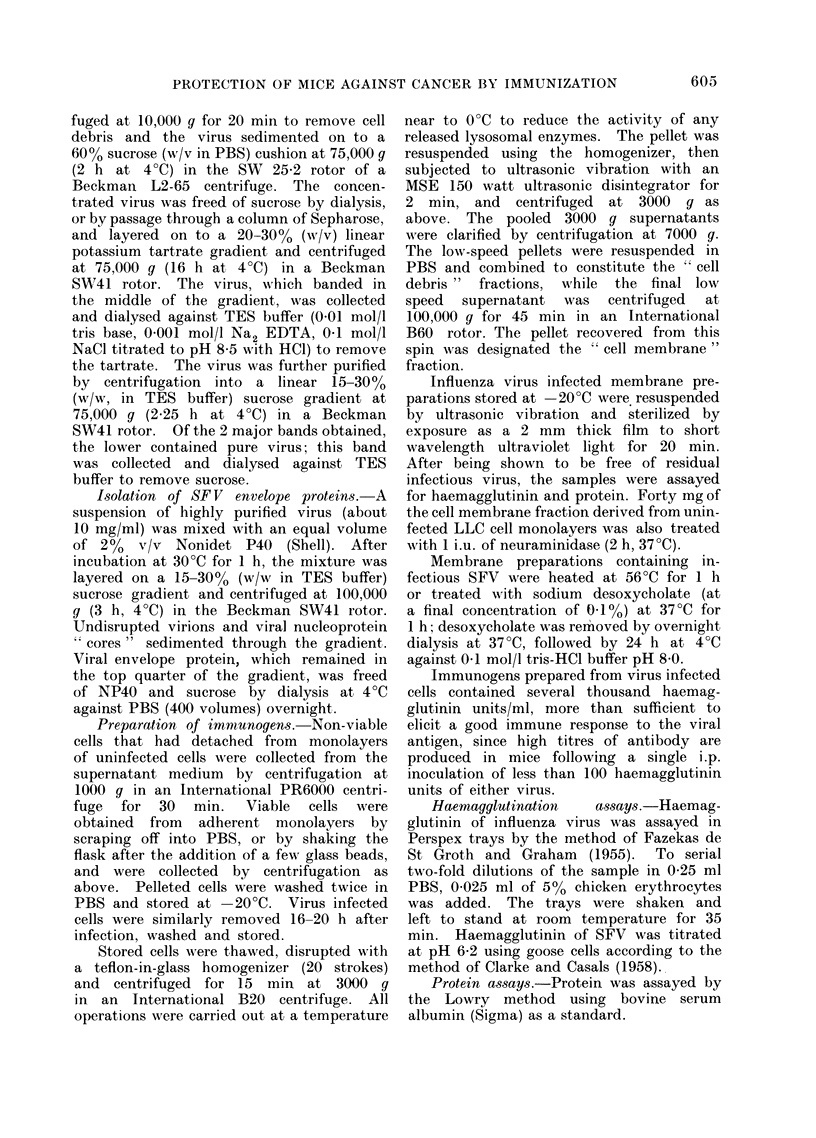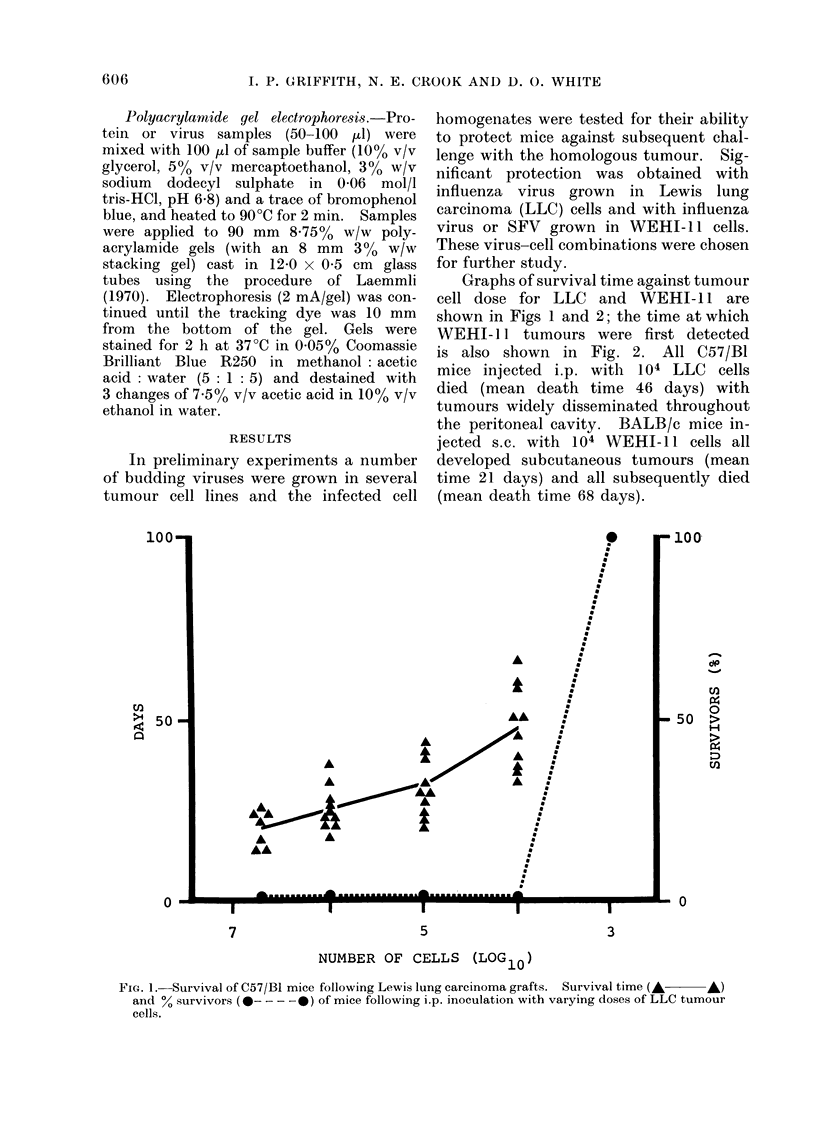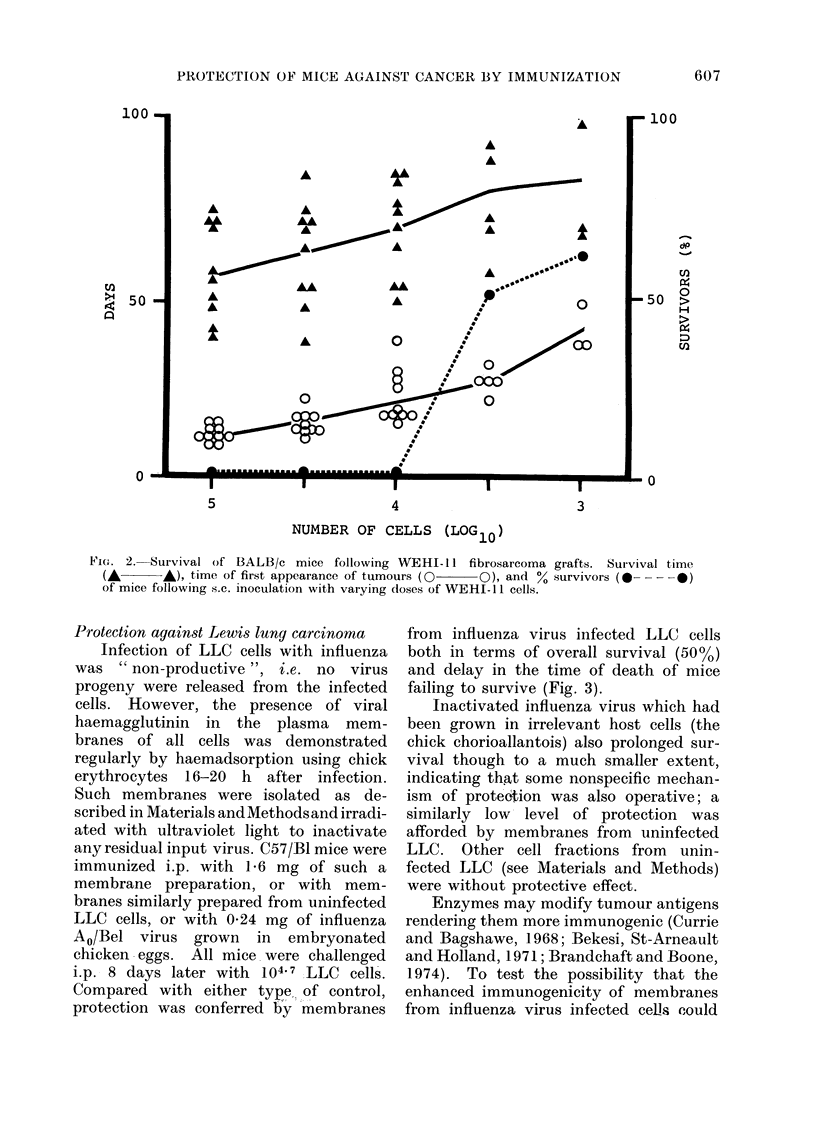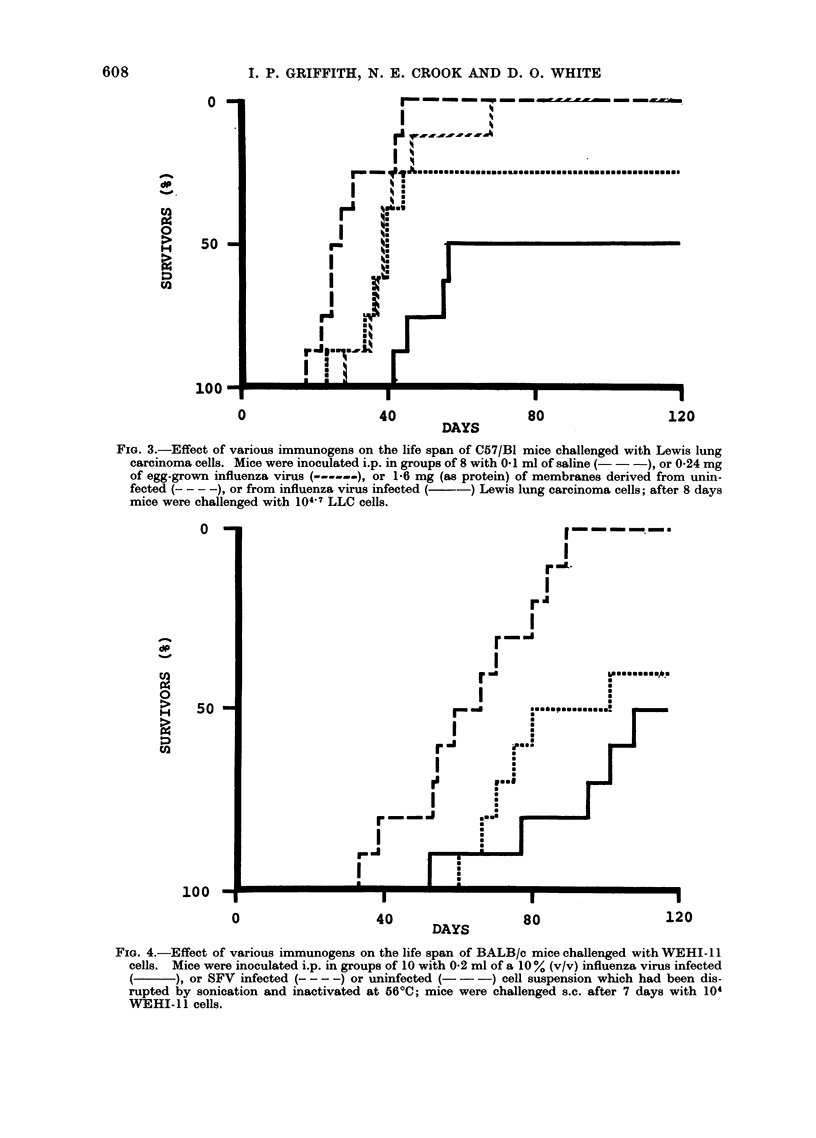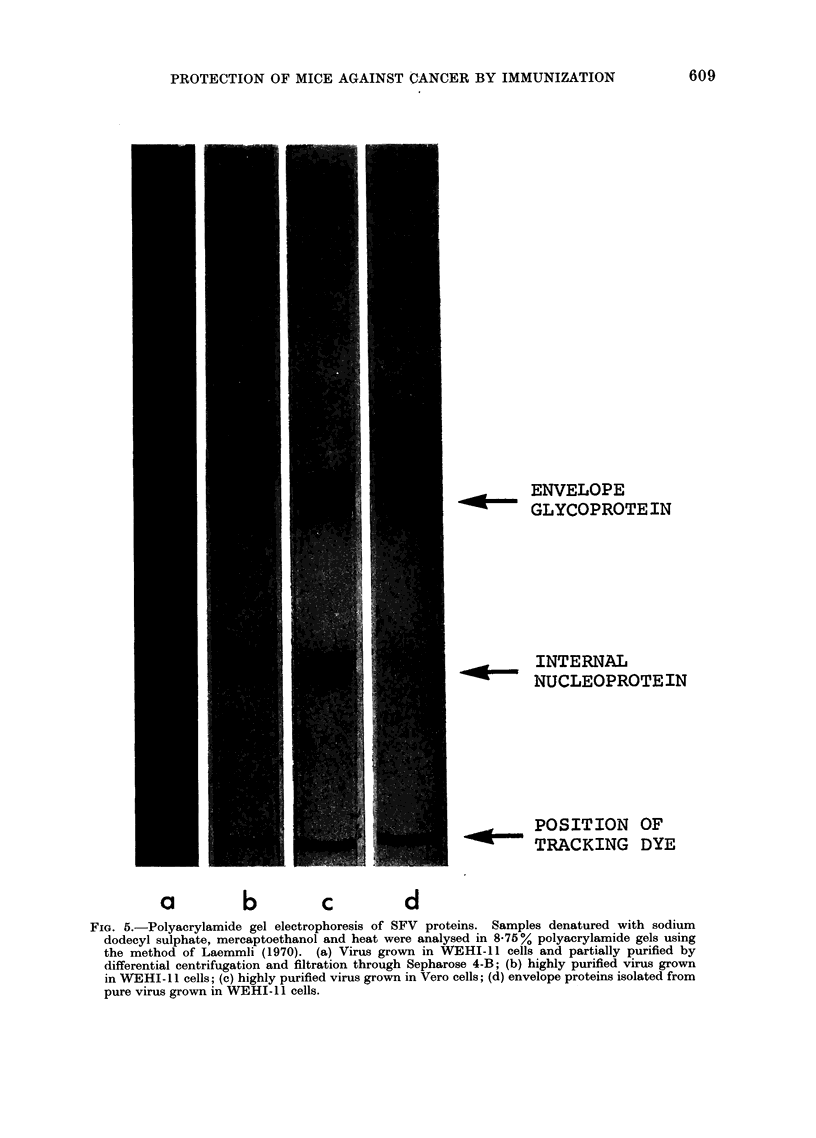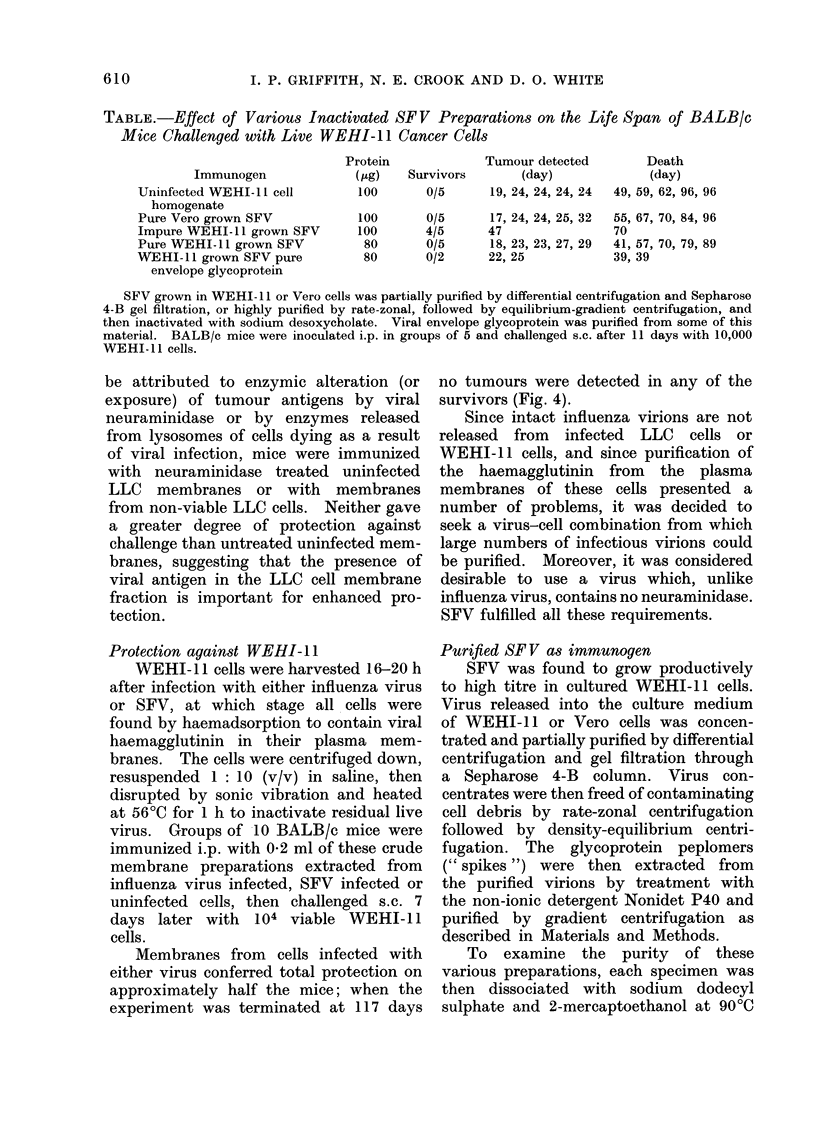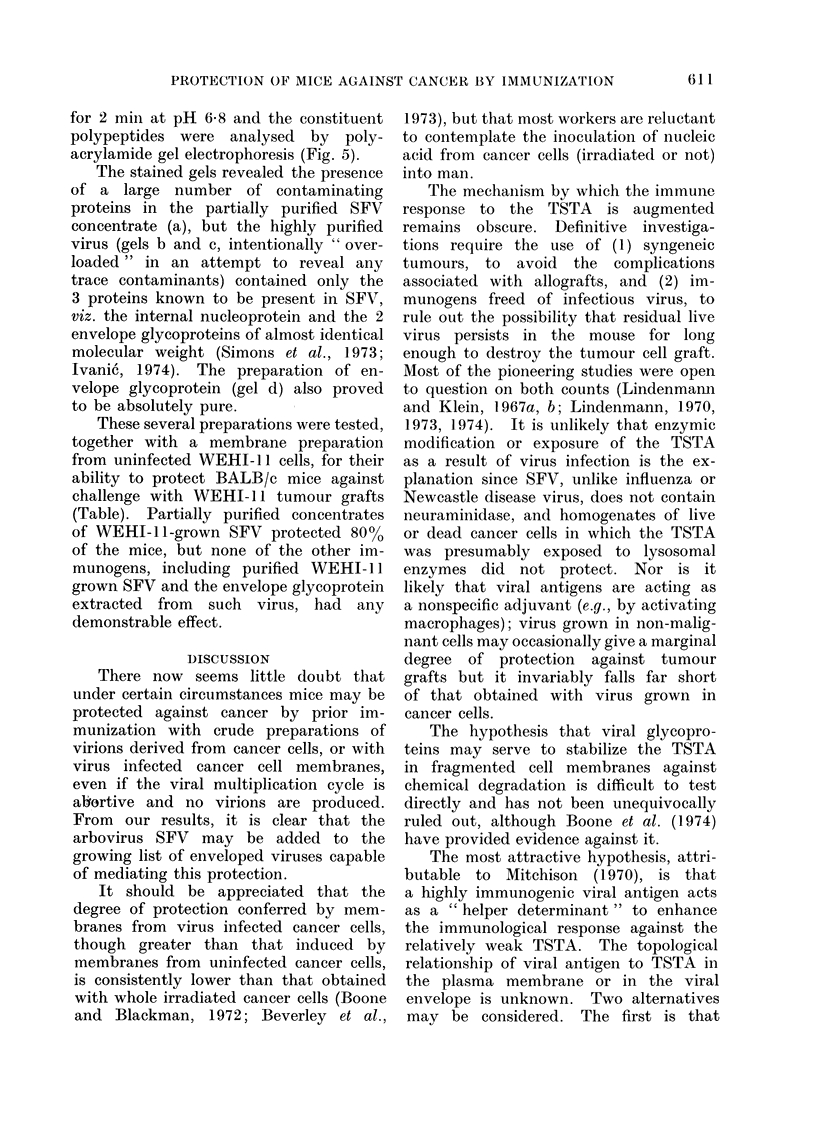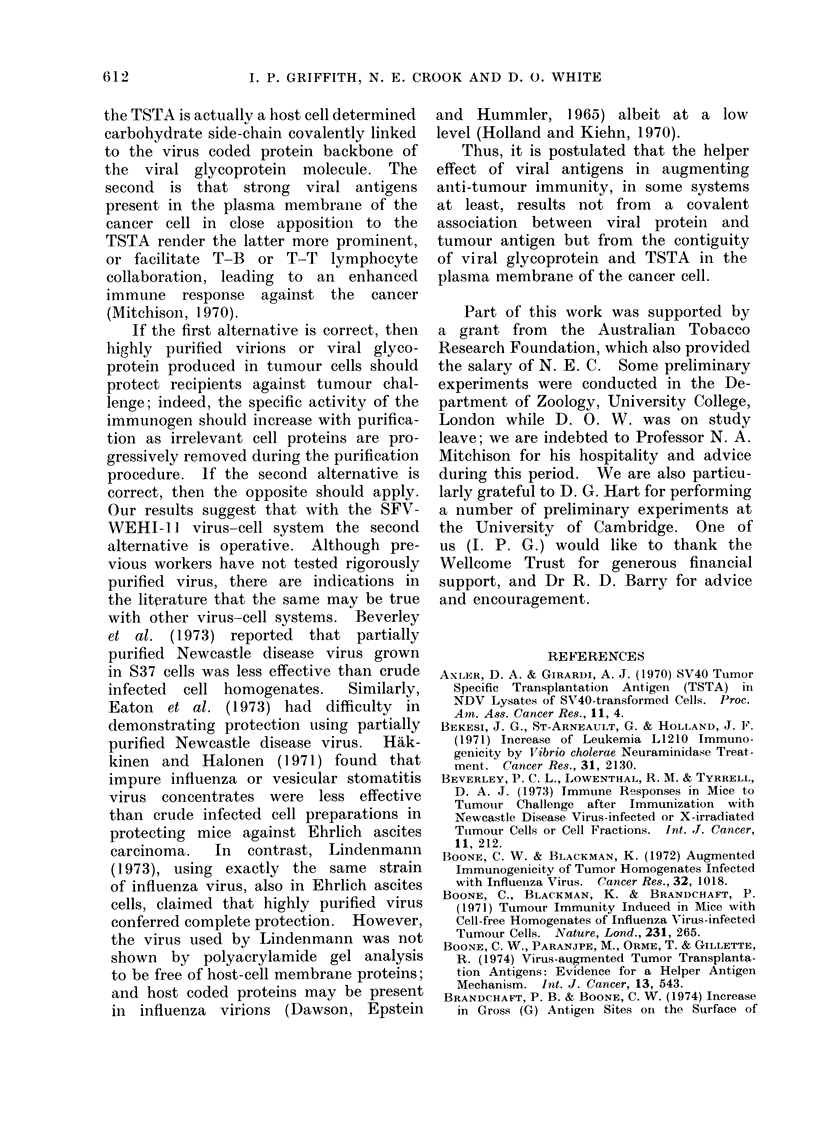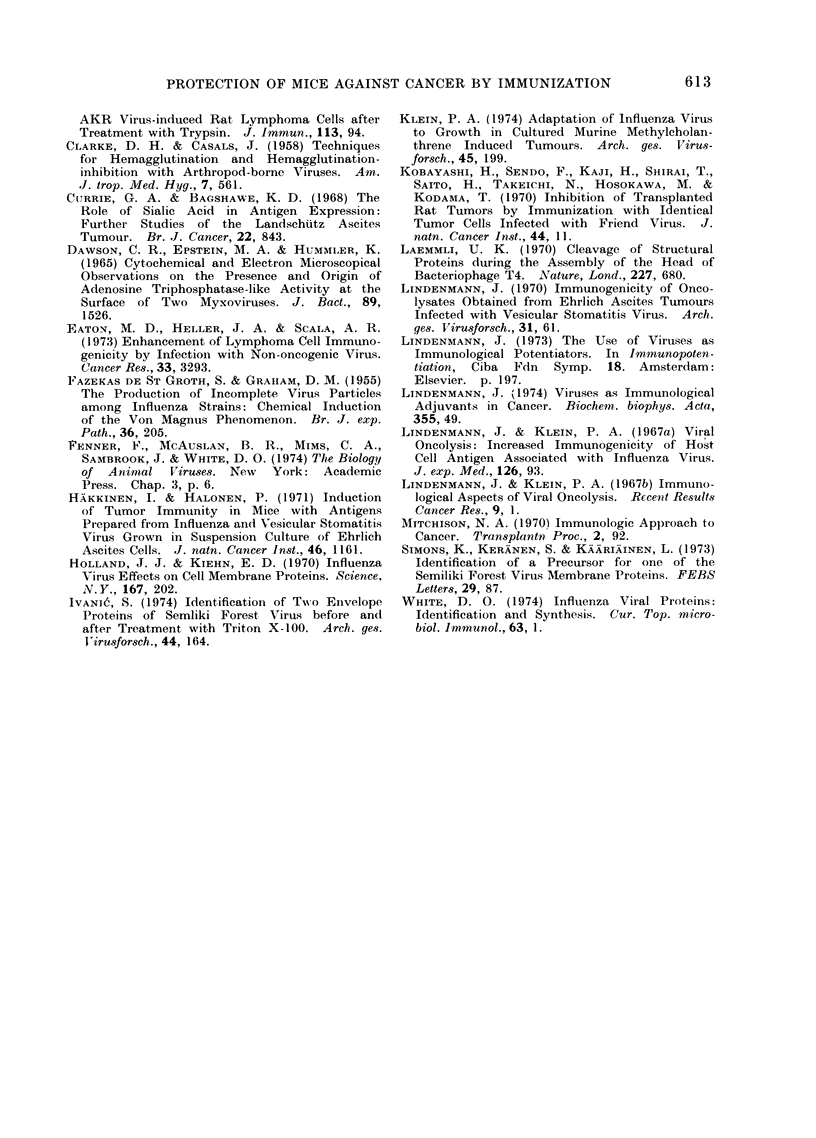# Protection of mice against cancer by immunization with membranes but not purified virions from virus infected cancer cells.

**DOI:** 10.1038/bjc.1975.106

**Published:** 1975-06

**Authors:** I. P. Griffith, N. E. Crook, D. O. White

## Abstract

**Images:**


*Br. J. Cancer* (1975) **31**, 603

### PROTECTION OF MICE AGAINST CANCER BY IMMUNIZATION WITH MEMBRANES BUT NOT PURIFIED VIRIONS FROM VIRUS INFECTED CANCER CELLS

I. P. GRIFFITH,* N. E. CROOK AND D. O. WHITE

*From the Division of Virology, Department of Pathology, University of Cambridge, England CB2 2QQ, and Department of Microbiology, University of Melbourne, Parkville, Victoria 3052, Australia*

Received 2 January 1975. Accepted 4 March 1975

**Summary.—The life span of C57/Bl mice inoculated with Lewis lung carcinoma cells was prolonged if the mice were pre-immunized with membranes from these cells infected *in vitro* with influenza virus. Likewise, BALB/c mice were protected against the malignant tumour WEHI-11 by prior immunization with extracts of cultured WEHI-11 cells which had been infected with influenza virus or Semliki Forest virus (SFV). Partially purified SFV grown in WEHI-11 cells also protected mice from cancer grafts but neither highly purified SFV nor the glycoprotein from the envelope of this virus protected the mice. It is concluded that SFV-induced immunopotentiation against cancer is not due to covalent linkage of tumour specific transplantation antigen (TSTA) to viral envelope protein but more probably is due to the apposition of viral glycoprotein and cellular TSTA in the plasma membrane of the cancer cell.**

THERE have been several reports that membranes from cancer cells infected with enveloped viruses protect mice or rats against tumour challenge more effectively than do membranes from uninfected cells. Viruses used include influenza strain WSA (Lindenmann and Klein, 1967*a*, *b*; Häkkinen and Halonen, 1971; Boone, Blackman and Brandchaft, 1971; Boone and Blackman, 1972; Boone *et al.*, 1974), influenza strain WSN (Klein, 1974), influenza $A_2$ Hongkong (Boone *et al.*, 1974), strains of avian influenza virus (Lindenmann and Klein, 1967*a*; Lindenmann, 1970), Newcastle disease virus (Axler and Girardi, 1970; Beverley, Lowenthal and Tyrrell, 1973; Eaton, Heller and Scala, 1973), vesicular stomatitis virus (Lindenmann, 1970; Häkkinen and Halonen, 1971; Boone *et al.*, 1974) and Friend leukaemia virus (Kobayashi *et al.*, 1970). Possible mechanisms of this virus induced immune potentiation have been discussed by Mitchison (1970), Lindenmann (1973, 1974) and Boone *et al.* (1974). One of the suggested mechanisms is that the viral antigen acts as a "helper determinant" (Mitchison, 1970).

This paper reports attempts to elucidate the spatial relationship between the hypothetical "helper determinant" and the tumour specific transplantation antigen (TSTA). A feature common to many, and probably all, budding viruses is that the virus coded envelope proteins of the virion are glycosylated by cell coded sugar transferases during virus synthesis in infected cells. Hence, the carbohydrate moiety of any virus coded glycoprotein is uniquely characteristic of the cell type in which that virus grew (see Fenner *et al.*, 1974). If, in virus infected cancer cells, the carbohydrate side-chain of a membrane glycoprotein were a critical determinant in the TSTA, it is possible that the TSTA could become incorporated into the viral glycoprotein. In this event highly purified virions grown in cancer cells (or even viral glycoprotein extracted from such virions) might be expected to protect mice from tumour challenge. To test this hypothesis we

* Present address and the one to which reprint requests should be directed: Victorian College of Pharmacy, Parkville, Victoria, 3052, Australia.

selected two enveloped viruses, the membrane glycoproteins of which may be purified by established procedures. Virions of influenza virus strain $A_0$/Bel contain only two glycoproteins in their envelopes, a neuraminidase and a haemagglutinin; the latter, which is present in greater amounts, may readily be purified (reviewed by White, 1974). Virions of Semliki Forest virus (SFV), a group A arbovirus (togavirus), are even simpler in their structure and also possess two envelope glycoproteins (Simons, Keränen and Kääriäinen, 1973; Ivanić, 1974).

### MATERIALS AND METHODS

*Virus*.—Viruses used were influenza, strain $A_0$/Bel, and Semliki Forest Virus (SFV).

*Mice*.—Young adult C57/Bl mice were from the colony held at the Department of Pathology, University of Cambridge, and BALB/c mice from the colony held at the Department of Microbiology, University of Melbourne.

*Tumour cell lines*.—Lewis lung carcinoma cells derived from C57/Bl mice were a gift from Dr N. Legge of the Imperial Cancer Research Fund, Lincoln's Inn Fields, London. WEHI-11, a fibrosarcoma of BALB/c mice, was kindly supplied by Dr N. Warner of the Walter and Eliza Hall Institute, Melbourne.

*Cell culture*.—Tumour cells were passed once in the appropriate host. Freshly excised tumours were finely minced with a scalpel blade or forced through an 80 gauge wire mesh. Minced tissue in serum-free Dulbecco's modified Eagle's medium (DMEM) was mixed with trypsin and EDTA to final concentrations of 0·25% and 0·02% respectively and stirred at 37°C. The cell suspension was decanted at 15-min intervals into an equal volume of calf serum and kept in ice; fresh medium containing trypsin–EDTA was added to the remaining tissue mince and the process repeated a number of times. Cells recovered from solid tumours in this way were washed once with phosphate buffered saline (PBS), resuspended in DMEM supplemented to 10% with foetal calf serum (FCS) and used immediately for cell culture, or were mixed with dimethylsulphoxide (to 10% v/v) and stored at −70°C or in liquid nitrogen.

Cells were cultured *in vitro* in flat glass bottles, or roller bottles, in DMEM containing 10% FCS, 0·03% glutamine, 0·01% streptomycin, 100 u/ml of penicillin G, $2{\cdot}5 \times 10^{-4}$ % Fungizone, 0·01 mol/l HEPES buffer and 0·17% sodium bicarbonate; the medium was adjusted to pH 7·3 with 1 N sodium hydroxide.

*Propagation and purification of virus*.—Influenza virus was propagated in the allantoic cavity of 11-day old embryonated hens' eggs. Eggs were inoculated with about 1000 "egg infectious doses" and incubated at 35°C for 40 h. The eggs were then chilled and infected allantoic fluids were harvested, clarified by low speed centrifugation, filtered through a washed 220 $\mu$m Millipore membrane, snap-frozen and stored at −70°C for stock virus.

To prepare concentrated influenza virus for vaccine, the infected allantoic fluid was cooled, clarified by low speed centrifugation and mixed with an equal volume of ammonium sulphate solution (neutralized with 1 N NaOH) saturated at 4°C. Flocculated virus was concentrated by centrifugation for 20 min at 8,000 *g* in an International B20 centrifuge. Following resuspension in PBS, the concentrated virus was centrifuged into a density gradient of glycerol/65% (w/w) sucrose/40% (w/v) potassium tartrate (in the ratio of 1 : 1 : 1, and diluted with 0·05% sodium azide to give a density range of 1·18–1·26 g/ml), for 2·5 h in a Beckmann L2 ultracentrifuge at 80,000 *g* in a SW27 rotor. The virus containing fraction was stored at 4°C. Before use glycerol, sucrose and tartrate were removed by passing the virus through a Sephadex G-50 column.

Propagation of influenza virus in tumour cell monolayers was carried out as follows. The monolayers were washed twice with PBS to remove serum, and cultured in DMEM medium, with 0·1% bovine serum albumin (Sigma) in place of FCS; virus was added at a multiplicity of 10 infectious particles/cell, and the cells incubated at 37°C for 16–20 h.

For the growth of SFV, virus was added to confluent WEHI-11 monolayers at an infectious virus to cell ratio of 1 : 10. After 24 h at 37°C the culture medium was centrifuged at 10,000 *g* for 20 min to remove cell debris and the virus sedimented on to a 60% sucrose (w/v in PBS) cushion at 75,000 *g* (2 h at 4°C) in the SW 25·2 rotor of a Beckman L2-65 centrifuge. The concentrated virus was freed of sucrose by dialysis, or by passage through a column of Sepharose, and layered on to a 20–30% (w/v) linear potassium tartrate gradient and centrifuged at 75,000 *g* (16 h at 4°C) in a Beckman SW41 rotor. The virus, which banded in the middle of the gradient, was collected and dialysed against TES buffer (0·01 mol/l tris base, 0·001 mol/l $Na_2$ EDTA, 0·1 mol/l NaCl titrated to pH 8·5 with HCl) to remove the tartrate. The virus was further purified by centrifugation into a linear 15–30% (w/w, in TES buffer) sucrose gradient at 75,000 *g* (2·25 h at 4°C) in a Beckman SW41 rotor. Of the 2 major bands obtained, the lower contained pure virus; this band was collected and dialysed against TES buffer to remove sucrose.

*Isolation of SFV envelope proteins.*—A suspension of highly purified virus (about 10 mg/ml) was mixed with an equal volume of 2% v/v Nonidet P40 (Shell). After incubation at 30°C for 1 h, the mixture was layered on a 15–30% (w/w in TES buffer) sucrose gradient and centrifuged at 100,000 *g* (3 h, 4°C) in the Beckman SW41 rotor. Undisrupted virions and viral nucleoprotein "cores" sedimented through the gradient. Viral envelope protein, which remained in the top quarter of the gradient, was freed of NP40 and sucrose by dialysis at 4°C against PBS (400 volumes) overnight.

*Preparation of immunogens.*—Non-viable cells that had detached from monolayers of uninfected cells were collected from the supernatant medium by centrifugation at 1000 *g* in an International PR6000 centrifuge for 30 min. Viable cells were obtained from adherent monolayers by scraping off into PBS, or by shaking the flask after the addition of a few glass beads, and were collected by centrifugation as above. Pelleted cells were washed twice in PBS and stored at −20°C. Virus infected cells were similarly removed 16–20 h after infection, washed and stored.

Stored cells were thawed, disrupted with a teflon-in-glass homogenizer (20 strokes) and centrifuged for 15 min at 3000 *g* in an International B20 centrifuge. All operations were carried out at a temperature near to 0°C to reduce the activity of any released lysosomal enzymes. The pellet was resuspended using the homogenizer, then subjected to ultrasonic vibration with an MSE 150 watt ultrasonic disintegrator for 2 min, and centrifuged at 3000 *g* as above. The pooled 3000 *g* supernatants were clarified by centrifugation at 7000 *g*. The low-speed pellets were resuspended in PBS and combined to constitute the "cell debris" fractions, while the final low speed supernatant was centrifuged at 100,000 *g* for 45 min in an International B60 rotor. The pellet recovered from this spin was designated the "cell membrane" fraction.

Influenza virus infected membrane preparations stored at −20°C were resuspended by ultrasonic vibration and sterilized by exposure as a 2 mm thick film to short wavelength ultraviolet light for 20 min. After being shown to be free of residual infectious virus, the samples were assayed for haemagglutinin and protein. Forty mg of the cell membrane fraction derived from uninfected LLC cell monolayers was also treated with 1 i.u. of neuraminidase (2 h, 37°C).

Membrane preparations containing infectious SFV were heated at 56°C for 1 h or treated with sodium desoxycholate (at a final concentration of 0·1%) at 37°C for 1 h; desoxycholate was removed by overnight dialysis at 37°C, followed by 24 h at 4°C against 0·1 mol/l tris-HCl buffer pH 8·0.

Immunogens prepared from virus infected cells contained several thousand haemagglutinin units/ml, more than sufficient to elicit a good immune response to the viral antigen, since high titres of antibody are produced in mice following a single i.p. inoculation of less than 100 haemagglutinin units of either virus.

*Haemagglutination assays.*—Haemagglutinin of influenza virus was assayed in Perspex trays by the method of Fazekas de St Groth and Graham (1955). To serial two-fold dilutions of the sample in 0·25 ml PBS, 0·025 ml of 5% chicken erythrocytes was added. The trays were shaken and left to stand at room temperature for 35 min. Haemagglutinin of SFV was titrated at pH 6·2 using goose cells according to the method of Clarke and Casals (1958).

*Protein assays.*—Protein was assayed by the Lowry method using bovine serum albumin (Sigma) as a standard.

*Polyacrylamide gel electrophoresis*.—Protein or virus samples (50–100 μl) were mixed with 100 μl of sample buffer (10% v/v glycerol, 5% v/v mercaptoethanol, 3% w/v sodium dodecyl sulphate in 0·06 mol/l tris-HCl, pH 6·8) and a trace of bromophenol blue, and heated to 90°C for 2 min. Samples were applied to 90 mm 8·75% w/w polyacrylamide gels (with an 8 mm 3% w/w stacking gel) cast in 12·0 × 0·5 cm glass tubes using the procedure of Laemmli (1970). Electrophoresis (2 mA/gel) was continued until the tracking dye was 10 mm from the bottom of the gel. Gels were stained for 2 h at 37°C in 0·05% Coomassie Brilliant Blue R250 in methanol : acetic acid : water (5 : 1 : 5) and destained with 3 changes of 7·5% v/v acetic acid in 10% v/v ethanol in water.

### RESULTS

In preliminary experiments a number of budding viruses were grown in several tumour cell lines and the infected cell homogenates were tested for their ability to protect mice against subsequent challenge with the homologous tumour. Significant protection was obtained with influenza virus grown in Lewis lung carcinoma (LLC) cells and with influenza virus or SFV grown in WEHI-11 cells. These virus–cell combinations were chosen for further study.

Graphs of survival time against tumour cell dose for LLC and WEHI-11 are shown in Figs 1 and 2; the time at which WEHI-11 tumours were first detected is also shown in Fig. 2. All C57/Bl mice injected i.p. with $10^4$ LLC cells died (mean death time 46 days) with tumours widely disseminated throughout the peritoneal cavity. BALB/c mice injected s.c. with $10^4$ WEHI-11 cells all developed subcutaneous tumours (mean time 21 days) and all subsequently died (mean death time 68 days).

Fig. 1.—Survival of C57/Bl mice following Lewis lung carcinoma grafts. Survival time (▲———▲) and % survivors (●– – – –●) of mice following i.p. inoculation with varying doses of LLC tumour cells.

FIG. 2.—Survival of BALB/c mice following WEHI-11 fibrosarcoma grafts. Survival time (▲———▲), time of first appearance of tumours (○———○), and % survivors (●- - - -●) of mice following s.c. inoculation with varying doses of WEHI-11 cells.

*Protection against Lewis lung carcinoma*

Infection of LLC cells with influenza was " non-productive ", *i.e.* no virus progeny were released from the infected cells. However, the presence of viral haemagglutinin in the plasma membranes of all cells was demonstrated regularly by haemadsorption using chick erythrocytes 16–20 h after infection. Such membranes were isolated as described in Materials and Methods and irradiated with ultraviolet light to inactivate any residual input virus. C57/Bl mice were immunized i.p. with 1·6 mg of such a membrane preparation, or with membranes similarly prepared from uninfected LLC cells, or with 0·24 mg of influenza $A_0$/Bel virus grown in embryonated chicken eggs. All mice were challenged i.p. 8 days later with $10^{4\cdot7}$ LLC cells. Compared with either type of control, protection was conferred by membranes from influenza virus infected LLC cells both in terms of overall survival (50%) and delay in the time of death of mice failing to survive (Fig. 3).

Inactivated influenza virus which had been grown in irrelevant host cells (the chick chorioallantois) also prolonged survival though to a much smaller extent, indicating that some nonspecific mechanism of protection was also operative; a similarly low level of protection was afforded by membranes from uninfected LLC. Other cell fractions from uninfected LLC (see Materials and Methods) were without protective effect.

Enzymes may modify tumour antigens rendering them more immunogenic (Currie and Bagshawe, 1968; Bekesi, St-Arneault and Holland, 1971; Brandchaft and Boone, 1974). To test the possibility that the enhanced immunogenicity of membranes from influenza virus infected cells could

FIG. 3.—Effect of various immunogens on the life span of C57/Bl mice challenged with Lewis lung carcinoma cells. Mice were inoculated i.p. in groups of 8 with 0·1 ml of saline (— — —), or 0·24 mg of egg-grown influenza virus (------), or 1·6 mg (as protein) of membranes derived from uninfected (- - - -), or from influenza virus infected (————) Lewis lung carcinoma cells; after 8 days mice were challenged with $10^{4\cdot7}$ LLC cells.

FIG. 4.—Effect of various immunogens on the life span of BALB/c mice challenged with WEHI-11 cells. Mice were inoculated i.p. in groups of 10 with 0·2 ml of a 10% (v/v) influenza virus infected (————), or SFV infected (- - - -) or uninfected (— — —) cell suspension which had been disrupted by sonication and inactivated at 56°C; mice were challenged s.c. after 7 days with $10^4$ WEHI-11 cells.

FIG. 5.—Polyacrylamide gel electrophoresis of SFV proteins. Samples denatured with sodium dodecyl sulphate, mercaptoethanol and heat were analysed in 8·75% polyacrylamide gels using the method of Laemmli (1970). (a) Virus grown in WEHI-11 cells and partially purified by differential centrifugation and filtration through Sepharose 4-B; (b) highly purified virus grown in WEHI-11 cells; (c) highly purified virus grown in Vero cells; (d) envelope proteins isolated from pure virus grown in WEHI-11 cells.

TABLE.—*Effect of Various Inactivated SFV Preparations on the Life Span of BALB/c Mice Challenged with Live WEHI-*11 *Cancer Cells*

| Immunogen | Protein ($\mu$g) | Survivors | Tumour detected (day) | Death (day) |
|---|---|---|---|---|
| Uninfected WEHI-11 cell homogenate | 100 | 0/5 | 19, 24, 24, 24, 24 | 49, 59, 62, 96, 96 |
| Pure Vero grown SFV | 100 | 0/5 | 17, 24, 24, 25, 32 | 55, 67, 70, 84, 96 |
| Impure WEHI-11 grown SFV | 100 | 4/5 | 47 | 70 |
| Pure WEHI-11 grown SFV | 80 | 0/5 | 18, 23, 23, 27, 29 | 41, 57, 70, 79, 89 |
| WEHI-11 grown SFV pure envelope glycoprotein | 80 | 0/2 | 22, 25 | 39, 39 |

SFV grown in WEHI-11 or Vero cells was partially purified by differential centrifugation and Sepharose 4-B gel filtration, or highly purified by rate-zonal, followed by equilibrium-gradient centrifugation, and then inactivated with sodium desoxycholate. Viral envelope glycoprotein was purified from some of this material. BALB/c mice were inoculated i.p. in groups of 5 and challenged s.c. after 11 days with 10,000 WEHI-11 cells.

be attributed to enzymic alteration (or exposure) of tumour antigens by viral neuraminidase or by enzymes released from lysosomes of cells dying as a result of viral infection, mice were immunized with neuraminidase treated uninfected LLC membranes or with membranes from non-viable LLC cells. Neither gave a greater degree of protection against challenge than untreated uninfected membranes, suggesting that the presence of viral antigen in the LLC cell membrane fraction is important for enhanced protection.

*Protection against WEHI*-11

WEHI-11 cells were harvested 16–20 h after infection with either influenza virus or SFV, at which stage all cells were found by haemadsorption to contain viral haemagglutinin in their plasma membranes. The cells were centrifuged down, resuspended 1 : 10 (v/v) in saline, then disrupted by sonic vibration and heated at 56°C for 1 h to inactivate residual live virus. Groups of 10 BALB/c mice were immunized i.p. with 0·2 ml of these crude membrane preparations extracted from influenza virus infected, SFV infected or uninfected cells, then challenged s.c. 7 days later with $10^4$ viable WEHI-11 cells.

Membranes from cells infected with either virus conferred total protection on approximately half the mice; when the experiment was terminated at 117 days no tumours were detected in any of the survivors (Fig. 4).

Since intact influenza virions are not released from infected LLC cells or WEHI-11 cells, and since purification of the haemagglutinin from the plasma membranes of these cells presented a number of problems, it was decided to seek a virus–cell combination from which large numbers of infectious virions could be purified. Moreover, it was considered desirable to use a virus which, unlike influenza virus, contains no neuraminidase. SFV fulfilled all these requirements.

*Purified SFV as immunogen*

SFV was found to grow productively to high titre in cultured WEHI-11 cells. Virus released into the culture medium of WEHI-11 or Vero cells was concentrated and partially purified by differential centrifugation and gel filtration through a Sepharose 4-B column. Virus concentrates were then freed of contaminating cell debris by rate-zonal centrifugation followed by density-equilibrium centrifugation. The glycoprotein peplomers ("spikes") were then extracted from the purified virions by treatment with the non-ionic detergent Nonidet P40 and purified by gradient centrifugation as described in Materials and Methods.

To examine the purity of these various preparations, each specimen was then dissociated with sodium dodecyl sulphate and 2-mercaptoethanol at 90°C

for 2 min at pH 6·8 and the constituent polypeptides were analysed by polyacrylamide gel electrophoresis (Fig. 5).

The stained gels revealed the presence of a large number of contaminating proteins in the partially purified SFV concentrate (a), but the highly purified virus (gels b and c, intentionally "overloaded" in an attempt to reveal any trace contaminants) contained only the 3 proteins known to be present in SFV, *viz.* the internal nucleoprotein and the 2 envelope glycoproteins of almost identical molecular weight (Simons *et al.*, 1973; Ivanić, 1974). The preparation of envelope glycoprotein (gel d) also proved to be absolutely pure.

These several preparations were tested, together with a membrane preparation from uninfected WEHI-11 cells, for their ability to protect BALB/c mice against challenge with WEHI-11 tumour grafts (Table). Partially purified concentrates of WEHI-11-grown SFV protected 80% of the mice, but none of the other immunogens, including purified WEHI-11 grown SFV and the envelope glycoprotein extracted from such virus, had any demonstrable effect.

### DISCUSSION

There now seems little doubt that under certain circumstances mice may be protected against cancer by prior immunization with crude preparations of virions derived from cancer cells, or with virus infected cancer cell membranes, even if the viral multiplication cycle is abortive and no virions are produced. From our results, it is clear that the arbovirus SFV may be added to the growing list of enveloped viruses capable of mediating this protection.

It should be appreciated that the degree of protection conferred by membranes from virus infected cancer cells, though greater than that induced by membranes from uninfected cancer cells, is consistently lower than that obtained with whole irradiated cancer cells (Boone and Blackman, 1972; Beverley *et al.*, 1973), but that most workers are reluctant to contemplate the inoculation of nucleic acid from cancer cells (irradiated or not) into man.

The mechanism by which the immune response to the TSTA is augmented remains obscure. Definitive investigations require the use of (1) syngeneic tumours, to avoid the complications associated with allografts, and (2) immunogens freed of infectious virus, to rule out the possibility that residual live virus persists in the mouse for long enough to destroy the tumour cell graft. Most of the pioneering studies were open to question on both counts (Lindenmann and Klein, 1967*a*, *b*; Lindenmann, 1970, 1973, 1974). It is unlikely that enzymic modification or exposure of the TSTA as a result of virus infection is the explanation since SFV, unlike influenza or Newcastle disease virus, does not contain neuraminidase, and homogenates of live or dead cancer cells in which the TSTA was presumably exposed to lysosomal enzymes did not protect. Nor is it likely that viral antigens are acting as a nonspecific adjuvant (*e.g.*, by activating macrophages); virus grown in non-malignant cells may occasionally give a marginal degree of protection against tumour grafts but it invariably falls far short of that obtained with virus grown in cancer cells.

The hypothesis that viral glycoproteins may serve to stabilize the TSTA in fragmented cell membranes against chemical degradation is difficult to test directly and has not been unequivocally ruled out, although Boone *et al.* (1974) have provided evidence against it.

The most attractive hypothesis, attributable to Mitchison (1970), is that a highly immunogenic viral antigen acts as a "helper determinant" to enhance the immunological response against the relatively weak TSTA. The topological relationship of viral antigen to TSTA in the plasma membrane or in the viral envelope is unknown. Two alternatives may be considered. The first is that the TSTA is actually a host cell determined carbohydrate side-chain covalently linked to the virus coded protein backbone of the viral glycoprotein molecule. The second is that strong viral antigens present in the plasma membrane of the cancer cell in close apposition to the TSTA render the latter more prominent, or facilitate T–B or T–T lymphocyte collaboration, leading to an enhanced immune response against the cancer (Mitchison, 1970).

If the first alternative is correct, then highly purified virions or viral glycoprotein produced in tumour cells should protect recipients against tumour challenge; indeed, the specific activity of the immunogen should increase with purification as irrelevant cell proteins are progressively removed during the purification procedure. If the second alternative is correct, then the opposite should apply. Our results suggest that with the SFV-WEHI-11 virus–cell system the second alternative is operative. Although previous workers have not tested rigorously purified virus, there are indications in the literature that the same may be true with other virus–cell systems. Beverley *et al.* (1973) reported that partially purified Newcastle disease virus grown in S37 cells was less effective than crude infected cell homogenates. Similarly, Eaton *et al.* (1973) had difficulty in demonstrating protection using partially purified Newcastle disease virus. Häkkinen and Halonen (1971) found that impure influenza or vesicular stomatitis virus concentrates were less effective than crude infected cell preparations in protecting mice against Ehrlich ascites carcinoma. In contrast, Lindenmann (1973), using exactly the same strain of influenza virus, also in Ehrlich ascites cells, claimed that highly purified virus conferred complete protection. However, the virus used by Lindenmann was not shown by polyacrylamide gel analysis to be free of host-cell membrane proteins; and host coded proteins may be present in influenza virions (Dawson, Epstein and Hummler, 1965) albeit at a low level (Holland and Kiehn, 1970).

Thus, it is postulated that the helper effect of viral antigens in augmenting anti-tumour immunity, in some systems at least, results not from a covalent association between viral protein and tumour antigen but from the contiguity of viral glycoprotein and TSTA in the plasma membrane of the cancer cell.

Part of this work was supported by a grant from the Australian Tobacco Research Foundation, which also provided the salary of N. E. C. Some preliminary experiments were conducted in the Department of Zoology, University College, London while D. O. W. was on study leave; we are indebted to Professor N. A. Mitchison for his hospitality and advice during this period. We are also particularly grateful to D. G. Hart for performing a number of preliminary experiments at the University of Cambridge. One of us (I. P. G.) would like to thank the Wellcome Trust for generous financial support, and Dr R. D. Barry for advice and encouragement.

### REFERENCES

Axler, D. A. & Girardi, A. J. (1970) SV40 Tumor Specific Transplantation Antigen (TSTA) in NDV Lysates of SV40-transformed Cells. *Proc. Am. Ass. Cancer Res.*, **11**, 4.

Bekesi, J. G., St-Arneault, G. & Holland, J. F. (1971) Increase of Leukemia L1210 Immunogenicity by *Vibrio cholerae* Neuraminidase Treatment. *Cancer Res.*, **31**, 2130.

Beverley, P. C. L., Lowenthal, R. M. & Tyrrell, D. A. J. (1973) Immune Responses in Mice to Tumour Challenge after Immunization with Newcastle Disease Virus-infected or X-irradiated Tumour Cells or Cell Fractions. *Int. J. Cancer*, **11**, 212.

Boone, C. W. & Blackman, K. (1972) Augmented Immunogenicity of Tumor Homogenates Infected with Influenza Virus. *Cancer Res.*, **32**, 1018.

Boone, C., Blackman, K. & Brandchaft, P. (1971) Tumour Immunity Induced in Mice with Cell-free Homogenates of Influenza Virus-infected Tumour Cells. *Nature, Lond.*, **231**, 265.

Boone, C. W., Paranjpe, M., Orme, T. & Gillette, R. (1974) Virus-augmented Tumor Transplantation Antigens: Evidence for a Helper Antigen Mechanism. *Int. J. Cancer*, **13**, 543.

Brandchaft, P. B. & Boone, C. W. (1974) Increase in Gross (G) Antigen Sites on the Surface of

AKR Virus-induced Rat Lymphoma Cells after Treatment with Trypsin. *J. Immun.*, **113**, 94.

Clarke, D. H. & Casals, J. (1958) Techniques for Hemagglutination and Hemagglutination-inhibition with Arthropod-borne Viruses. *Am. J. trop. Med. Hyg.*, **7**, 561.

Currie, G. A. & Bagshawe, K. D. (1968) The Role of Sialic Acid in Antigen Expression: Further Studies of the Landschütz Ascites Tumour. *Br. J. Cancer*, **22**, 843.

Dawson, C. R., Epstein, M. A. & Hummler, K. (1965) Cytochemical and Electron Microscopical Observations on the Presence and Origin of Adenosine Triphosphatase-like Activity at the Surface of Two Myxoviruses. *J. Bact.*, **89**, 1526.

Eaton, M. D., Heller, J. A. & Scala, A. R. (1973) Enhancement of Lymphoma Cell Immunogenicity by Infection with Non-oncogenic Virus. *Cancer Res.*, **33**, 3293.

Fazekas de St Groth, S. & Graham, D. M. (1955) The Production of Incomplete Virus Particles among Influenza Strains: Chemical Induction of the Von Magnus Phenomenon. *Br. J. exp. Path.*, **36**, 205.

Fenner, F., McAuslan, B. R., Mims, C. A., Sambrook, J. & White, D. O. (1974) *The Biology of Animal Viruses.* New York: Academic Press. Chap. 3, p. 6.

Häkkinen, I. & Halonen, P. (1971) Induction of Tumor Immunity in Mice with Antigens Prepared from Influenza and Vesicular Stomatitis Virus Grown in Suspension Culture of Ehrlich Ascites Cells. *J. natn. Cancer Inst.*, **46**, 1161.

Holland, J. J. & Kiehn, E. D. (1970) Influenza Virus Effects on Cell Membrane Proteins. *Science, N.Y.*, **167**, 202.

Ivanić, S. (1974) Identification of Two Envelope Proteins of Semliki Forest Virus before and after Treatment with Triton X-100. *Arch. ges. Virusforsch.*, **44**, 164.

Klein, P. A. (1974) Adaptation of Influenza Virus to Growth in Cultured Murine Methylcholanthrene Induced Tumours. *Arch. ges. Virusforsch.*, **45**, 199.

Kobayashi, H., Sendo, F., Kaji, H., Shirai, T., Saito, H., Takeichi, N., Hosokawa, M. & Kodama, T. (1970) Inhibition of Transplanted Rat Tumors by Immunization with Identical Tumor Cells Infected with Friend Virus. *J. natn. Cancer Inst.*, **44**, 11.

Laemmli, U. K. (1970) Cleavage of Structural Proteins during the Assembly of the Head of Bacteriophage T4. *Nature, Lond.*, **227**, 680.

Lindenmann, J. (1970) Immunogenicity of Oncolysates Obtained from Ehrlich Ascites Tumours Infected with Vesicular Stomatitis Virus. *Arch. ges. Virusforsch.*, **31**, 61.

Lindenmann, J. (1973) The Use of Viruses as Immunological Potentiators. In *Immunopotentiation*, Ciba Fdn Symp. **18**. Amsterdam: Elsevier. p. 197.

Lindenmann, J. (1974) Viruses as Immunological Adjuvants in Cancer. *Biochem. biophys. Acta*, **355**, 49.

Lindenmann, J. & Klein, P. A. (1967*a*) Viral Oncolysis: Increased Immunogenicity of Host Cell Antigen Associated with Influenza Virus. *J. exp. Med.*, **126**, 93.

Lindenmann, J. & Klein, P. A. (1967*b*) Immunological Aspects of Viral Oncolysis. *Recent Results Cancer Res.*, **9**, 1.

Mitchison, N. A. (1970) Immunologic Approach to Cancer. *Transplantn Proc.*, **2**, 92.

Simons, K., Keränen, S. & Kääriäinen, L. (1973) Identification of a Precursor for one of the Semiliki Forest Virus Membrane Proteins. *FEBS Letters*, **29**, 87.

White, D. O. (1974) Influenza Viral Proteins: Identification and Synthesis. *Cur. Top. microbiol. Immunol.*, **63**, 1.